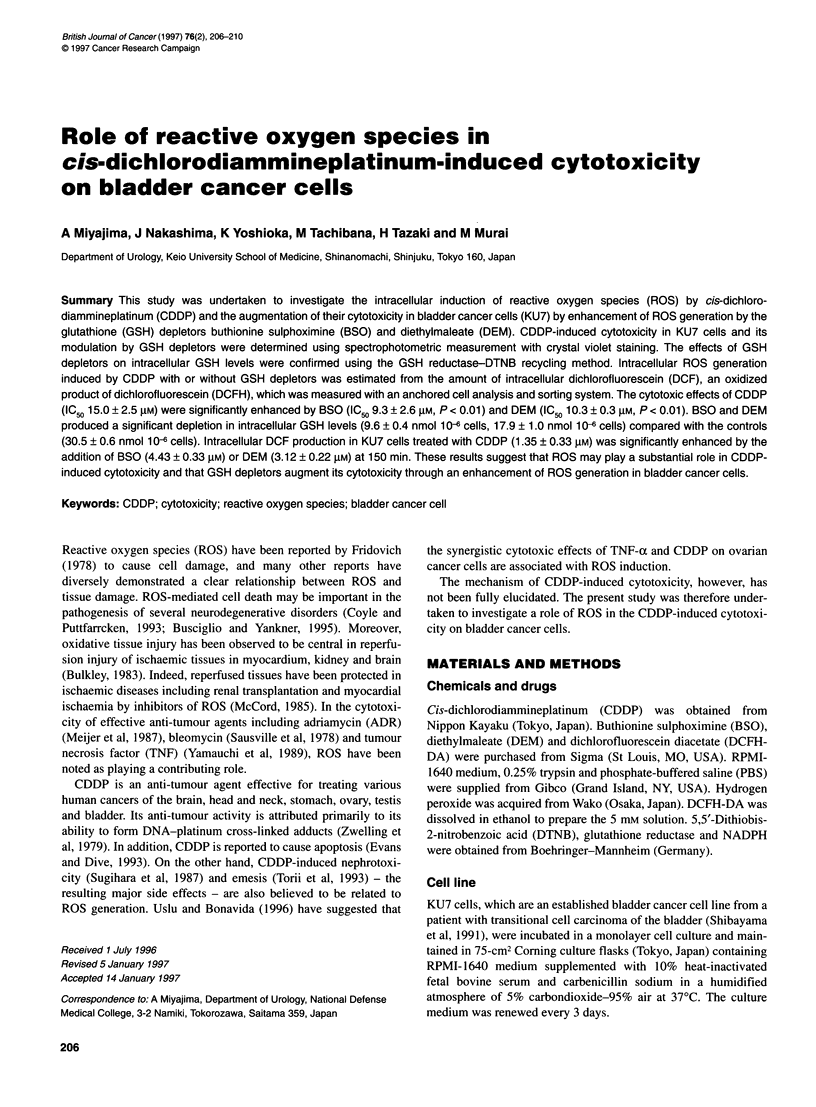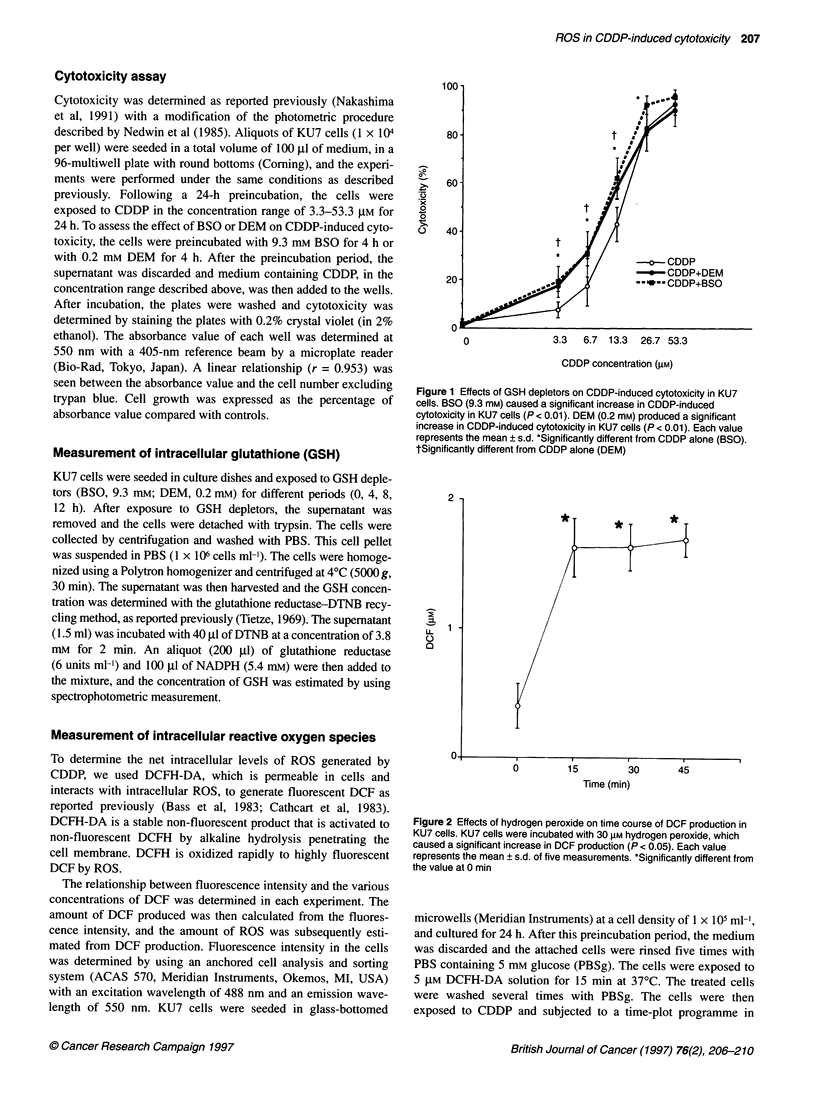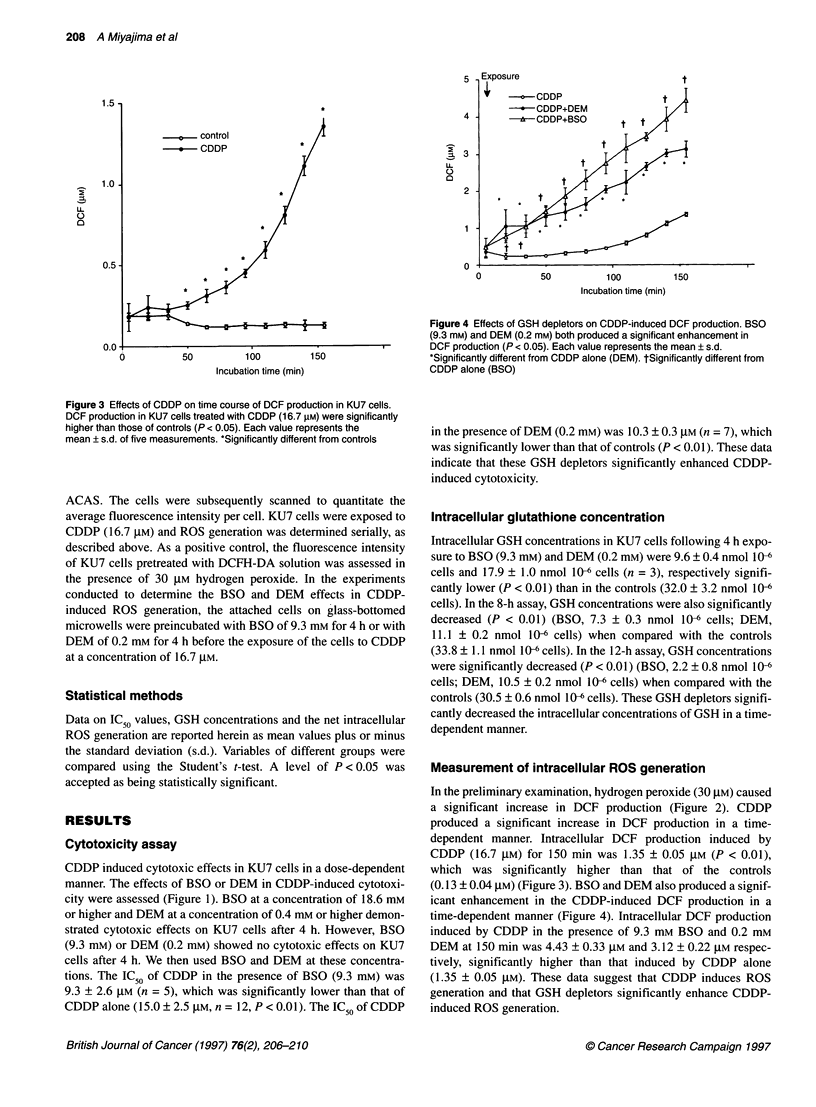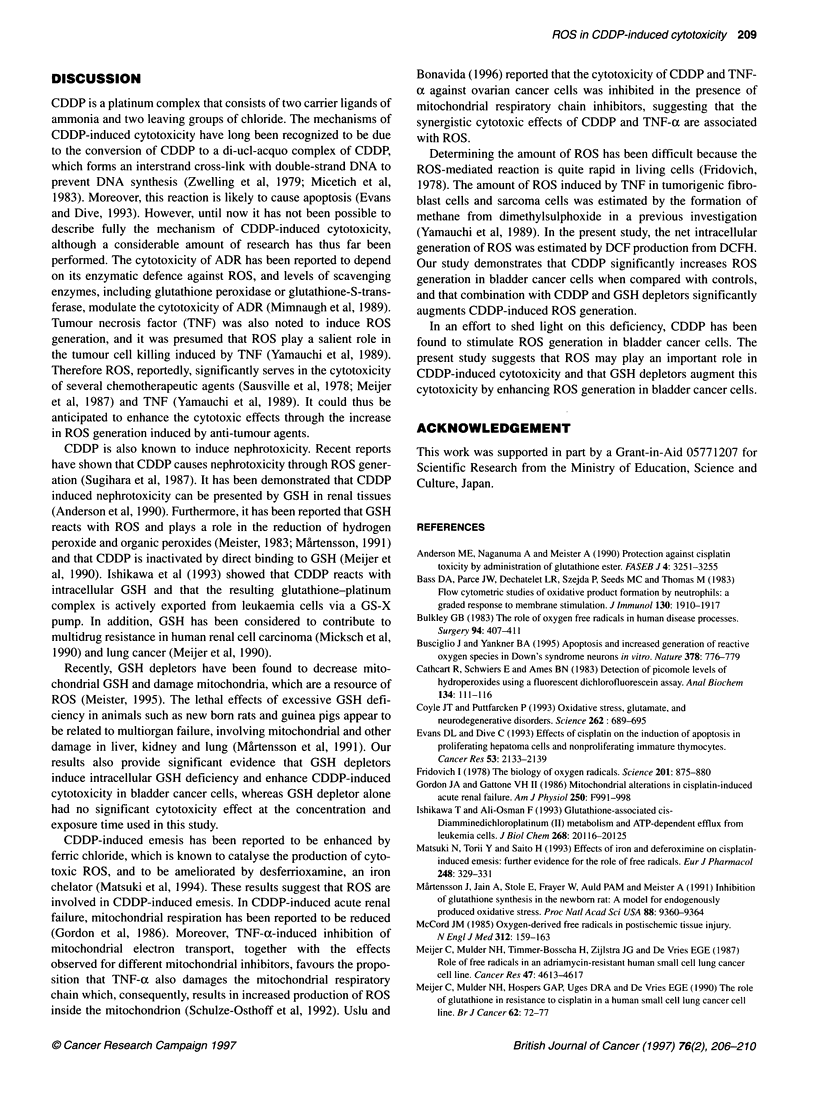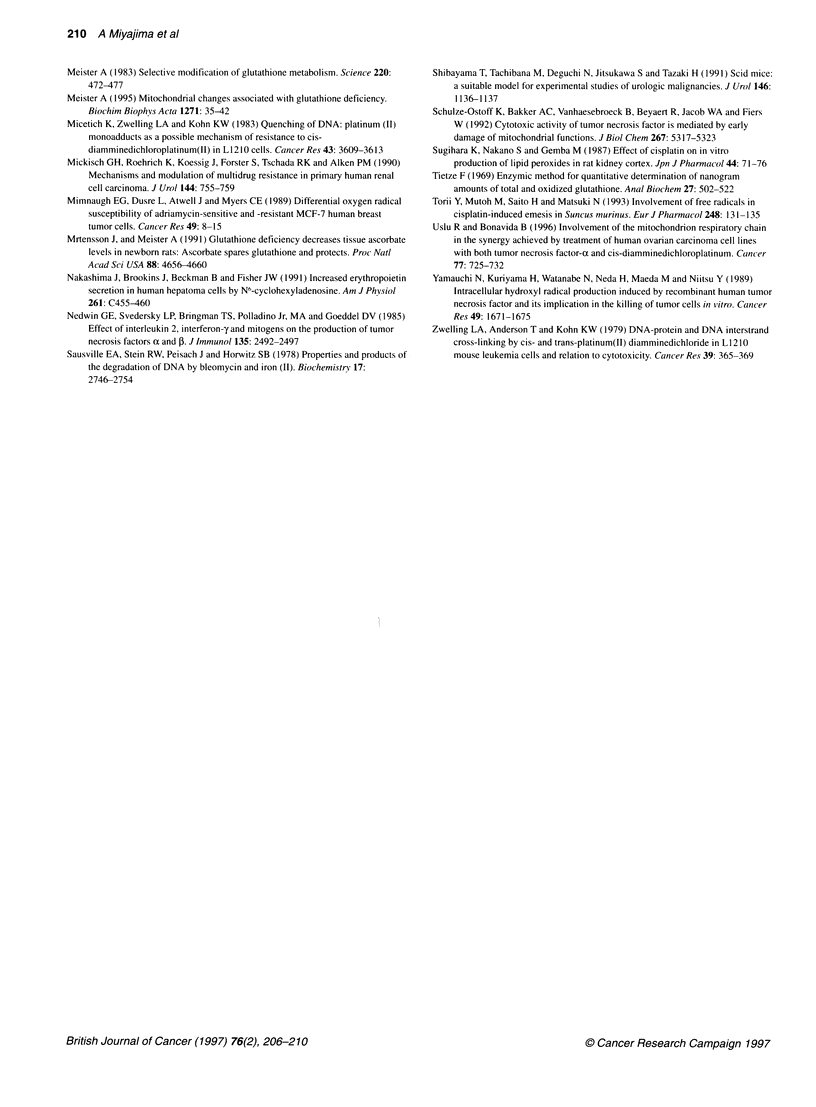# Role of reactive oxygen species in cis-dichlorodiammineplatinum-induced cytotoxicity on bladder cancer cells.

**DOI:** 10.1038/bjc.1997.363

**Published:** 1997

**Authors:** A. Miyajima, J. Nakashima, K. Yoshioka, M. Tachibana, H. Tazaki, M. Murai

**Affiliations:** Department of Urology, Keio University School of Medicine, Shinjuku, Tokyo, Japan.

## Abstract

This study was undertaken to investigate the intracellular induction of reactive oxygen species (ROS) by cis-dichlorodiammineplatinum (CDDP) and the augmentation of their cytotoxicity in bladder cancer cells (KU7) by enhancement of ROS generation by the glutathione (GSH) depletors buthionine sulphoximine (BSO) and diethylmaleate (DEM). CDDP-induced cytotoxicity in KU7 cells and its modulation by GSH depletors were determined using spectrophotometric measurement with crystal violet staining. The effects of GSH depletors on intracellular GSH levels were confirmed using the GSH reductase-DTNB recycling method. Intracellular ROS generation induced by CDDP with or without GSH depletors was estimated from the amount of intracellular dichlorofluorescein (DCF), an oxidized product of dichlorofluorescein (DCFH), which was measured with an anchored cell analysis and sorting system. The cytotoxic effects of CDDP (IC50 15.0 +/- 2.5 microM) were significantly enhanced by BSO (IC50 9.3 +/- 2.6 microM, P < 0.01) and DEM (IC50 10.3 +/- 0.3 microM, P <0.01). BSO and DEM produced a significant depletion in intracellular GSH levels (9.6 +/- 0.4 nmol 10(-6) cells, 17.9 +/- 1.0 nmol 10(-6) cells) compared with the controls (30.5 +/- 0.6 nmol 10(-6) cells). Intracellular DCF production in KU7 cells treated with CDDP (1.35 +/- 0.33 microM) was significantly enhanced by the addition of BSO (4.43 +/- 0.33 microM) or DEM (3.12 +/- 0.22 microM) at 150 min. These results suggest that ROS may play a substantial role in CDDP-induced cytotoxicity and that GSH depletors augment its cytotoxicity through an enhancement of ROS generation in bladder cancer cells.


British Journal of Cancer (1997) 76(2), 206–210
© 1997 Cancer Research Campaign

### Role of reactive oxygen species in *cis*-dichlorodiammineplatinum-induced cytotoxicity on bladder cancer cells

**A Miyajima, J Nakashima, K Yoshioka, M Tachibana, H Tazaki and M Murai**

Department of Urology, Keio University School of Medicine, Shinanomachi, Shinjuku, Tokyo 160, Japan

**Summary** This study was undertaken to investigate the intracellular induction of reactive oxygen species (ROS) by *cis*-dichlorodiammineplatinum (CDDP) and the augmentation of their cytotoxicity in bladder cancer cells (KU7) by enhancement of ROS generation by the glutathione (GSH) depletors buthionine sulphoximine (BSO) and diethylmaleate (DEM). CDDP-induced cytotoxicity in KU7 cells and its modulation by GSH depletors were determined using spectrophotometric measurement with crystal violet staining. The effects of GSH depletors on intracellular GSH levels were confirmed using the GSH reductase–DTNB recycling method. Intracellular ROS generation induced by CDDP with or without GSH depletors was estimated from the amount of intracellular dichlorofluorescein (DCF), an oxidized product of dichlorofluorescein (DCFH), which was measured with an anchored cell analysis and sorting system. The cytotoxic effects of CDDP ($IC_{50}$ 15.0 ± 2.5 μM) were significantly enhanced by BSO ($IC_{50}$ 9.3 ± 2.6 μM, $P < 0.01$) and DEM ($IC_{50}$ 10.3 ± 0.3 μM, $P < 0.01$). BSO and DEM produced a significant depletion in intracellular GSH levels (9.6 ± 0.4 nmol $10^{-6}$ cells, 17.9 ± 1.0 nmol $10^{-6}$ cells) compared with the controls (30.5 ± 0.6 nmol $10^{-6}$ cells). Intracellular DCF production in KU7 cells treated with CDDP (1.35 ± 0.33 μM) was significantly enhanced by the addition of BSO (4.43 ± 0.33 μM) or DEM (3.12 ± 0.22 μM) at 150 min. These results suggest that ROS may play a substantial role in CDDP-induced cytotoxicity and that GSH depletors augment its cytotoxicity through an enhancement of ROS generation in bladder cancer cells.

**Keywords:** CDDP; cytotoxicity; reactive oxygen species; bladder cancer cell

Reactive oxygen species (ROS) have been reported by Fridovich (1978) to cause cell damage, and many other reports have diversely demonstrated a clear relationship between ROS and tissue damage. ROS-mediated cell death may be important in the pathogenesis of several neurodegenerative disorders (Coyle and Puttfarrcken, 1993; Busciglio and Yankner, 1995). Moreover, oxidative tissue injury has been observed to be central in reperfusion injury of ischaemic tissues in myocardium, kidney and brain (Bulkley, 1983). Indeed, reperfused tissues have been protected in ischaemic diseases including renal transplantation and myocardial ischaemia by inhibitors of ROS (McCord, 1985). In the cytotoxicity of effective anti-tumour agents including adriamycin (ADR) (Meijer et al, 1987), bleomycin (Sausville et al, 1978) and tumour necrosis factor (TNF) (Yamauchi et al, 1989), ROS have been noted as playing a contributing role.

CDDP is an anti-tumour agent effective for treating various human cancers of the brain, head and neck, stomach, ovary, testis and bladder. Its anti-tumour activity is attributed primarily to its ability to form DNA–platinum cross-linked adducts (Zwelling et al, 1979). In addition, CDDP is reported to cause apoptosis (Evans and Dive, 1993). On the other hand, CDDP-induced nephrotoxicity (Sugihara et al, 1987) and emesis (Torii et al, 1993) – the resulting major side effects – are also believed to be related to ROS generation. Uslu and Bonavida (1996) have suggested that the synergistic cytotoxic effects of TNF-α and CDDP on ovarian cancer cells are associated with ROS induction.

The mechanism of CDDP-induced cytotoxicity, however, has not been fully elucidated. The present study was therefore undertaken to investigate a role of ROS in the CDDP-induced cytotoxicity on bladder cancer cells.

*Received 1 July 1996*
*Revised 5 January 1997*
*Accepted 14 January 1997*

*Correspondence to:* A Miyajima, Department of Urology, National Defense Medical College, 3-2 Namiki, Tokorozawa, Saitama 359, Japan

### MATERIALS AND METHODS

### Chemicals and drugs

*Cis*-dichlorodiammineplatinum (CDDP) was obtained from Nippon Kayaku (Tokyo, Japan). Buthionine sulphoximine (BSO), diethylmaleate (DEM) and dichlorofluorescein diacetate (DCFH-DA) were purchased from Sigma (St Louis, MO, USA). RPMI-1640 medium, 0.25% trypsin and phosphate-buffered saline (PBS) were supplied from Gibco (Grand Island, NY, USA). Hydrogen peroxide was acquired from Wako (Osaka, Japan). DCFH-DA was dissolved in ethanol to prepare the 5 mM solution. 5,5′-Dithiobis-2-nitrobenzoic acid (DTNB), glutathione reductase and NADPH were obtained from Boehringer–Mannheim (Germany).

### Cell line

KU7 cells, which are an established bladder cancer cell line from a patient with transitional cell carcinoma of the bladder (Shibayama et al, 1991), were incubated in a monolayer cell culture and maintained in 75-cm² Corning culture flasks (Tokyo, Japan) containing RPMI-1640 medium supplemented with 10% heat-inactivated fetal bovine serum and carbenicillin sodium in a humidified atmosphere of 5% carbondioxide–95% air at 37°C. The culture medium was renewed every 3 days.

### Cytotoxicity assay

Cytotoxicity was determined as reported previously (Nakashima et al, 1991) with a modification of the photometric procedure described by Nedwin et al (1985). Aliquots of KU7 cells ($1 \times 10^4$ per well) were seeded in a total volume of 100 µl of medium, in a 96-multiwell plate with round bottoms (Corning), and the experiments were performed under the same conditions as described previously. Following a 24-h preincubation, the cells were exposed to CDDP in the concentration range of 3.3–53.3 µM for 24 h. To assess the effect of BSO or DEM on CDDP-induced cytotoxicity, the cells were preincubated with 9.3 mM BSO for 4 h or with 0.2 mM DEM for 4 h. After the preincubation period, the supernatant was discarded and medium containing CDDP, in the concentration range described above, was then added to the wells. After incubation, the plates were washed and cytotoxicity was determined by staining the plates with 0.2% crystal violet (in 2% ethanol). The absorbance value of each well was determined at 550 nm with a 405-nm reference beam by a microplate reader (Bio-Rad, Tokyo, Japan). A linear relationship ($r$ = 0.953) was seen between the absorbance value and the cell number excluding trypan blue. Cell growth was expressed as the percentage of absorbance value compared with controls.

### Measurement of intracellular glutathione (GSH)

KU7 cells were seeded in culture dishes and exposed to GSH depletors (BSO, 9.3 mM; DEM, 0.2 mM) for different periods (0, 4, 8, 12 h). After exposure to GSH depletors, the supernatant was removed and the cells were detached with trypsin. The cells were collected by centrifugation and washed with PBS. This cell pellet was suspended in PBS ($1 \times 10^6$ cells $ml^{-1}$). The cells were homogenized using a Polytron homogenizer and centrifuged at 4°C (5000 *g*, 30 min). The supernatant was then harvested and the GSH concentration was determined with the glutathione reductase–DTNB recycling method, as reported previously (Tietze, 1969). The supernatant (1.5 ml) was incubated with 40 µl of DTNB at a concentration of 3.8 mM for 2 min. An aliquot (200 µl) of glutathione reductase (6 units $ml^{-1}$) and 100 µl of NADPH (5.4 mM) were then added to the mixture, and the concentration of GSH was estimated by using spectrophotometric measurement.

### Measurement of intracellular reactive oxygen species

To determine the net intracellular levels of ROS generated by CDDP, we used DCFH-DA, which is permeable in cells and interacts with intracellular ROS, to generate fluorescent DCF as reported previously (Bass et al, 1983; Cathcart et al, 1983). DCFH-DA is a stable non-fluorescent product that is activated to non-fluorescent DCFH by alkaline hydrolysis penetrating the cell membrane. DCFH is oxidized rapidly to highly fluorescent DCF by ROS.

The relationship between fluorescence intensity and the various concentrations of DCF was determined in each experiment. The amount of DCF produced was then calculated from the fluorescence intensity, and the amount of ROS was subsequently estimated from DCF production. Fluorescence intensity in the cells was determined by using an anchored cell analysis and sorting system (ACAS 570, Meridian Instruments, Okemos, MI, USA) with an excitation wavelength of 488 nm and an emission wavelength of 550 nm. KU7 cells were seeded in glass-bottomed microwells (Meridian Instruments) at a cell density of $1 \times 10^5$ $ml^{-1}$, and cultured for 24 h. After this preincubation period, the medium was discarded and the attached cells were rinsed five times with PBS containing 5 mM glucose (PBSg). The cells were exposed to 5 µM DCFH-DA solution for 15 min at 37°C. The treated cells were washed several times with PBSg. The cells were then exposed to CDDP and subjected to a time-plot programme in

**Figure 1** Effects of GSH depletors on CDDP-induced cytotoxicity in KU7 cells. BSO (9.3 mM) caused a significant increase in CDDP-induced cytotoxicity in KU7 cells ($P < 0.01$). DEM (0.2 mM) produced a significant increase in CDDP-induced cytotoxicity in KU7 cells ($P < 0.01$). Each value represents the mean ± s.d. *Significantly different from CDDP alone (BSO). †Significantly different from CDDP alone (DEM)

**Figure 2** Effects of hydrogen peroxide on time course of DCF production in KU7 cells. KU7 cells were incubated with 30 µM hydrogen peroxide, which caused a significant increase in DCF production ($P < 0.05$). Each value represents the mean ± s.d. of five measurements. *Significantly different from the value at 0 min

**Figure 3** Effects of CDDP on time course of DCF production in KU7 cells. DCF production in KU7 cells treated with CDDP (16.7 μM) were significantly higher than those of controls ($P < 0.05$). Each value represents the mean ± s.d. of five measurements. *Significantly different from controls

**Figure 4** Effects of GSH depletors on CDDP-induced DCF production. BSO (9.3 mM) and DEM (0.2 mM) both produced a significant enhancement in DCF production ($P < 0.05$). Each value represents the mean ± s.d. *Significantly different from CDDP alone (DEM). †Significantly different from CDDP alone (BSO)

ACAS. The cells were subsequently scanned to quantitate the average fluorescence intensity per cell. KU7 cells were exposed to CDDP (16.7 μM) and ROS generation was determined serially, as described above. As a positive control, the fluorescence intensity of KU7 cells pretreated with DCFH-DA solution was assessed in the presence of 30 μM hydrogen peroxide. In the experiments conducted to determine the BSO and DEM effects in CDDP-induced ROS generation, the attached cells on glass-bottomed microwells were preincubated with BSO of 9.3 mM for 4 h or with DEM of 0.2 mM for 4 h before the exposure of the cells to CDDP at a concentration of 16.7 μM.

### Statistical methods

Data on $IC_{50}$ values, GSH concentrations and the net intracellular ROS generation are reported herein as mean values plus or minus the standard deviation (s.d.). Variables of different groups were compared using the Student's *t*-test. A level of $P < 0.05$ was accepted as being statistically significant.

### RESULTS

### Cytotoxicity assay

CDDP induced cytotoxic effects in KU7 cells in a dose-dependent manner. The effects of BSO or DEM in CDDP-induced cytotoxicity were assessed (Figure 1). BSO at a concentration of 18.6 mM or higher and DEM at a concentration of 0.4 mM or higher demonstrated cytotoxic effects on KU7 cells after 4 h. However, BSO (9.3 mM) or DEM (0.2 mM) showed no cytotoxic effects on KU7 cells after 4 h. We then used BSO and DEM at these concentrations. The $IC_{50}$ of CDDP in the presence of BSO (9.3 mM) was 9.3 ± 2.6 μM ($n = 5$), which was significantly lower than that of CDDP alone (15.0 ± 2.5 μM, $n = 12$, $P < 0.01$). The $IC_{50}$ of CDDP in the presence of DEM (0.2 mM) was 10.3 ± 0.3 μM ($n = 7$), which was significantly lower than that of controls ($P < 0.01$). These data indicate that these GSH depletors significantly enhanced CDDP-induced cytotoxicity.

### Intracellular glutathione concentration

Intracellular GSH concentrations in KU7 cells following 4 h exposure to BSO (9.3 mM) and DEM (0.2 mM) were 9.6 ± 0.4 nmol $10^{-6}$ cells and 17.9 ± 1.0 nmol $10^{-6}$ cells ($n = 3$), respectively significantly lower ($P < 0.01$) than in the controls (32.0 ± 3.2 nmol $10^{-6}$ cells). In the 8-h assay, GSH concentrations were also significantly decreased ($P < 0.01$) (BSO, 7.3 ± 0.3 nmol $10^{-6}$ cells; DEM, 11.1 ± 0.2 nmol $10^{-6}$ cells) when compared with the controls (33.8 ± 1.1 nmol $10^{-6}$ cells). In the 12-h assay, GSH concentrations were significantly decreased ($P < 0.01$) (BSO, 2.2 ± 0.8 nmol $10^{-6}$ cells; DEM, 10.5 ± 0.2 nmol $10^{-6}$ cells) when compared with the controls (30.5 ± 0.6 nmol $10^{-6}$ cells). These GSH depletors significantly decreased the intracellular concentrations of GSH in a time-dependent manner.

### Measurement of intracellular ROS generation

In the preliminary examination, hydrogen peroxide (30 μM) caused a significant increase in DCF production (Figure 2). CDDP produced a significant increase in DCF production in a time-dependent manner. Intracellular DCF production induced by CDDP (16.7 μM) for 150 min was 1.35 ± 0.05 μM ($P < 0.01$), which was significantly higher than that of the controls (0.13 ± 0.04 μM) (Figure 3). BSO and DEM also produced a significant enhancement in the CDDP-induced DCF production in a time-dependent manner (Figure 4). Intracellular DCF production induced by CDDP in the presence of 9.3 mM BSO and 0.2 mM DEM at 150 min was 4.43 ± 0.33 μM and 3.12 ± 0.22 μM respectively, significantly higher than that induced by CDDP alone (1.35 ± 0.05 μM). These data suggest that CDDP induces ROS generation and that GSH depletors significantly enhance CDDP-induced ROS generation.

© Cancer Research Campaign 1997

### DISCUSSION

CDDP is a platinum complex that consists of two carrier ligands of ammonia and two leaving groups of chloride. The mechanisms of CDDP-induced cytotoxicity have long been recognized to be due to the conversion of CDDP to a di-ucl-acquo complex of CDDP, which forms an interstrand cross-link with double-strand DNA to prevent DNA synthesis (Zwelling et al, 1979; Micetich et al, 1983). Moreover, this reaction is likely to cause apoptosis (Evans and Dive, 1993). However, until now it has not been possible to describe fully the mechanism of CDDP-induced cytotoxicity, although a considerable amount of research has thus far been performed. The cytotoxicity of ADR has been reported to depend on its enzymatic defence against ROS, and levels of scavenging enzymes, including glutathione peroxidase or glutathione-S-transferase, modulate the cytotoxicity of ADR (Mimnaugh et al, 1989). Tumour necrosis factor (TNF) was also noted to induce ROS generation, and it was presumed that ROS play a salient role in the tumour cell killing induced by TNF (Yamauchi et al, 1989). Therefore ROS, reportedly, significantly serves in the cytotoxicity of several chemotherapeutic agents (Sausville et al, 1978; Meijer et al, 1987) and TNF (Yamauchi et al, 1989). It could thus be anticipated to enhance the cytotoxic effects through the increase in ROS generation induced by anti-tumour agents.

CDDP is also known to induce nephrotoxicity. Recent reports have shown that CDDP causes nephrotoxicity through ROS generation (Sugihara et al, 1987). It has been demonstrated that CDDP induced nephrotoxicity can be presented by GSH in renal tissues (Anderson et al, 1990). Furthermore, it has been reported that GSH reacts with ROS and plays a role in the reduction of hydrogen peroxide and organic peroxides (Meister, 1983; Mårtensson, 1991) and that CDDP is inactivated by direct binding to GSH (Meijer et al, 1990). Ishikawa et al (1993) showed that CDDP reacts with intracellular GSH and that the resulting glutathione–platinum complex is actively exported from leukaemia cells via a GS-X pump. In addition, GSH has been considered to contribute to multidrug resistance in human renal cell carcinoma (Micksch et al, 1990) and lung cancer (Meijer et al, 1990).

Recently, GSH depletors have been found to decrease mitochondrial GSH and damage mitochondria, which are a resource of ROS (Meister, 1995). The lethal effects of excessive GSH deficiency in animals such as new born rats and guinea pigs appear to be related to multiorgan failure, involving mitochondrial and other damage in liver, kidney and lung (Mårtensson et al, 1991). Our results also provide significant evidence that GSH depletors induce intracellular GSH deficiency and enhance CDDP-induced cytotoxicity in bladder cancer cells, whereas GSH depletor alone had no significant cytotoxicity effect at the concentration and exposure time used in this study.

CDDP-induced emesis has been reported to be enhanced by ferric chloride, which is known to catalyse the production of cytotoxic ROS, and to be ameliorated by desferrioxamine, an iron chelator (Matsuki et al, 1994). These results suggest that ROS are involved in CDDP-induced emesis. In CDDP-induced acute renal failure, mitochondrial respiration has been reported to be reduced (Gordon et al, 1986). Moreover, TNF-α-induced inhibition of mitochondrial electron transport, together with the effects observed for different mitochondrial inhibitors, favours the proposition that TNF-α also damages the mitochondrial respiratory chain which, consequently, results in increased production of ROS inside the mitochondrion (Schulze-Osthoff et al, 1992). Uslu and Bonavida (1996) reported that the cytotoxicity of CDDP and TNF-α against ovarian cancer cells was inhibited in the presence of mitochondrial respiratory chain inhibitors, suggesting that the synergistic cytotoxic effects of CDDP and TNF-α are associated with ROS.

Determining the amount of ROS has been difficult because the ROS-mediated reaction is quite rapid in living cells (Fridovich, 1978). The amount of ROS induced by TNF in tumorigenic fibroblast cells and sarcoma cells was estimated by the formation of methane from dimethylsulphoxide in a previous investigation (Yamauchi et al, 1989). In the present study, the net intracellular generation of ROS was estimated by DCF production from DCFH. Our study demonstrates that CDDP significantly increases ROS generation in bladder cancer cells when compared with controls, and that combination with CDDP and GSH depletors significantly augments CDDP-induced ROS generation.

In an effort to shed light on this deficiency, CDDP has been found to stimulate ROS generation in bladder cancer cells. The present study suggests that ROS may play an important role in CDDP-induced cytotoxicity and that GSH depletors augment this cytotoxicity by enhancing ROS generation in bladder cancer cells.

### ACKNOWLEDGEMENT

This work was supported in part by a Grant-in-Aid 05771207 for Scientific Research from the Ministry of Education, Science and Culture, Japan.

### REFERENCES

Anderson ME, Naganuma A and Meister A (1990) Protection against cisplatin toxicity by administration of glutathione ester. *FASEB J* **4**: 3251–3255

Bass DA, Parce JW, Dechatelet LR, Szejda P, Seeds MC and Thomas M (1983) Flow cytometric studies of oxidative product formation by neutrophils: a graded response to membrane stimulation. *J Immunol* **130**: 1910–1917

Bulkley GB (1983) The role of oxygen free radicals in human disease processes. *Surgery* **94**: 407–411

Busciglio J and Yankner BA (1995) Apoptosis and increased generation of reactive oxygen species in Down's syndrome neurons *in vitro*. *Nature* **378**: 776–779

Cathcart R, Schwiers E and Ames BN (1983) Detection of picomole levels of hydroperoxides using a fluorescent dichlorofluorescein assay. *Anal Biochem* **134**: 111–116

Coyle JT and Puttfarcken P (1993) Oxidative stress, glutamate, and neurodegenerative disorders. *Science* **262** : 689–695

Evans DL and Dive C (1993) Effects of cisplatin on the induction of apoptosis in proliferating hepatoma cells and nonproliferating immature thymocytes. *Cancer Res* **53**: 2133–2139

Fridovich I (1978) The biology of oxygen radicals. *Science* **201**: 875–880

Gordon JA and Gattone VH II (1986) Mitochondrial alterations in cisplatin-induced acute renal failure. *Am J Physiol* **250**: F991–998

Ishikawa T and Ali-Osman F (1993) Glutathione-associated cis-Diamminedichloroplatinum (II) metabolism and ATP-dependent efflux from leukemia cells. *J Biol Chem* **268**: 20116–20125

Matsuki N, Torii Y and Saito H (1993) Effects of iron and deferoximine on cisplatin-induced emesis: further evidence for the role of free radicals. *Eur J Pharmacol* **248**: 329–331

Mårtensson J, Jain A, Stole E, Frayer W, Auld PAM and Meister A (1991) Inhibition of glutathione synthesis in the newborn rat: A model for endogenously produced oxidative stress. *Proc Natl Acad Sci USA* **88**: 9360–9364

McCord JM (1985) Oxygen-derived free radicals in postischemic tissue injury. *N Engl J Med* **312**: 159–163

Meijer C, Mulder NH, Timmer-Bosscha H, Zijlstra JG and De Vries EGE (1987) Role of free radicals in an adriamycin-resistant human small cell lung cancer cell line. *Cancer Res* **47**: 4613–4617

Meijer C, Mulder NH, Hospers GAP, Uges DRA and De Vries EGE (1990) The role of glutathione in resistance to cisplatin in a human small cell lung cancer cell line. *Br J Cancer* **62**: 72–77

© Cancer Research Campaign 1997 

Meister A (1983) Selective modification of glutathione metabolism. *Science* **220**: 472–477

Meister A (1995) Mitochondrial changes associated with glutathione deficiency. *Biochim Biophys Acta* **1271**: 35–42

Micetich K, Zwelling LA and Kohn KW (1983) Quenching of DNA: platinum (II) monoadducts as a possible mechanism of resistance to cis-diamminedichloroplatinum(II) in L1210 cells. *Cancer Res* **43**: 3609–3613

Mickisch GH, Roehrich K, Koessig J, Forster S, Tschada RK and Alken PM (1990) Mechanisms and modulation of multidrug resistance in primary human renal cell carcinoma. *J Urol* **144**: 755–759

Mimnaugh EG, Dusre L, Atwell J and Myers CE (1989) Differential oxygen radical susceptibility of adriamycin-sensitive and -resistant MCF-7 human breast tumor cells. *Cancer Res* **49**: 8–15

Mrtensson J, and Meister A (1991) Glutathione deficiency decreases tissue ascorbate levels in newborn rats: Ascorbate spares glutathione and protects. *Proc Natl Acad Sci USA* **88**: 4656–4660

Nakashima J, Brookins J, Beckman B and Fisher JW (1991) Increased erythropoietin secretion in human hepatoma cells by $N^6$-cyclohexyladenosine. *Am J Physiol* **261**: C455–460

Nedwin GE, Svedersky LP, Bringman TS, Polladino Jr, MA and Goeddel DV (1985) Effect of interleukin 2, interferon-γ and mitogens on the production of tumor necrosis factors α and β. *J Immunol* **135**: 2492–2497

Sausville EA, Stein RW, Peisach J and Horwitz SB (1978) Properties and products of the degradation of DNA by bleomycin and iron (II). *Biochemistry* **17**: 2746–2754

Shibayama T, Tachibana M, Deguchi N, Jitsukawa S and Tazaki H (1991) Scid mice: a suitable model for experimental studies of urologic malignancies. *J Urol* **146**: 1136–1137

Schulze-Ostoff K, Bakker AC, Vanhaesebroeck B, Beyaert R, Jacob WA and Fiers W (1992) Cytotoxic activity of tumor necrosis factor is mediated by early damage of mitochondrial functions. *J Biol Chem* **267**: 5317–5323

Sugihara K, Nakano S and Gemba M (1987) Effect of cisplatin on in vitro production of lipid peroxides in rat kidney cortex. *Jpn J Pharmacol* **44**: 71–76

Tietze F (1969) Enzymic method for quantitative determination of nanogram amounts of total and oxidized glutathione. *Anal Biochem* **27**: 502–522

Torii Y, Mutoh M, Saito H and Matsuki N (1993) Involvement of free radicals in cisplatin-induced emesis in *Suncus murinus*. *Eur J Pharmacol* **248**: 131–135

Uslu R and Bonavida B (1996) Involvement of the mitochondrion respiratory chain in the synergy achieved by treatment of human ovarian carcinoma cell lines with both tumor necrosis factor-α and cis-diamminedichloroplatinum. *Cancer* **77**: 725–732

Yamauchi N, Kuriyama H, Watanabe N, Neda H, Maeda M and Niitsu Y (1989) Intracellular hydroxyl radical production induced by recombinant human tumor necrosis factor and its implication in the killing of tumor cells *in vitro*. *Cancer Res* **49**: 1671–1675

Zwelling LA, Anderson T and Kohn KW (1979) DNA-protein and DNA interstrand cross-linking by cis- and trans-platinum(II) diamminedichloride in L1210 mouse leukemia cells and relation to cytotoxicity. *Cancer Res* **39**: 365–369

© Cancer Research Campaign 1997